# Systematic review and network meta-analysis of effects of noninvasive brain stimulation on post-stroke cognitive impairment

**DOI:** 10.3389/fnins.2022.1082383

**Published:** 2022-12-28

**Authors:** Yueying Wang, Ning Xu, Runfang Wang, Weiyi Zai

**Affiliations:** College of Rehabilitation Medicine, Shandong University of Traditional Chinese Medicine, Jinan, China

**Keywords:** noninvasive brain stimulation (NIBS), transcranial direct current stimulation (tDCS), transcranial magnetic stimulation (TMS), post stroke cognitive impairment (PSCI), systematic review, meta-analysis

## Abstract

**Objective:**

To systematically assess the effects of Noninvasive Brain Stimulation (NIBS) on post-stroke cognitive impairment (PSCI) and to compare the efficacy of two different NIBS.

**Methods:**

Computer searches of PubMed, Web of Science, Cochrane Library, Embase, China National Knowledge Infrastructure (CNKI), China Science and Technology Journal Database (VIP), Chinese Biomedical literature Service System (SinoMed), and Wanfang Database were conducted using a combination of free words and subject terms. The search was conducted from the database creation date to 27 November 2022. The risk of bias in the included literature was assessed using the Cochrane Risk Assessment Scale. The quality of the included literature was assessed using the physiotherapy evidence database (PEDro) scale. A standard meta-analysis of study data for each outcome indicator was performed using RevMan 5.4 software. Network meta-analysis was performed using State 14.0 according to the Bayesian framework.

**Results:**

A total of 18 studies involving 809 patients were included. Meta-analysis shows NIBS significantly improved montreal cognitive assessment (MoCA) scores (standardized mean difference [SMD] = 0.76, 95% confidence interval (CI) 0.49–1.02, *P* < 0.05), mini-mental state examination (MMSE) scores (SMD = 0.72, 95% CI 0.25–1.20, *P* < 0.05), and modified barthel index (MBI) and functional independence measurement (FIM) scores (SMD = 0.33, 95% CI 0.11–0.54, *P* < 0.05) in patients with PSCI. The surface under the cumulative ranking curve (SUCRA) of different NIBS in improving MoCA scores were in the order of transcranial direct current stimulation (tDCS) (SUCRA = 92.4%) and transcranial magnetic stimulation (TMS) (SUCRA = 57.6%). The SUCRA of different NIBS in improving MMSE scores were in the order of tDCS (SUCRA = 81.6%) and TMS (SUCRA = 67.3%). The SUCRA of different NIBS in improving MBI and FIM scores were in the order of tDCS (SUCRA = 78.6%) and TMS (SUCRA = 65.3%).

**Conclusion:**

The available evidence suggests that NIBS improves cognitive impairment. tDCS appeared more effective than TMS for cognitive function and activities of daily living in PSCI patients. Limited by the number of included studies, more large-sample, multicentre, double-blind, high-quality randomized controlled clinical trials are needed to further confirm this study's results.

**Systematic review registration:**

https://www.crd.york.ac.uk/prospero/, identifier: CRD42022372354.

## Introduction

Stroke is a neurological disorder caused by blood circulation disorder and is the second leading cause of death worldwide, with over 13 million new cases each year (Feigin et al., 2022). Studies have shown that the incidence of post-stroke cognitive impairment (PSCI) is 80.97%, significantly affecting patients' ability to care for themselves and participate in society (Qu et al., 2015; Du et al., 2020; Weaver et al., 2021). Therefore, the rehabilitation of cognitive function in stroke patients is an issue that requires urgent attention. Currently, the treatment for patients with PSCI consists of medication and cognitive rehabilitation training. However, there are problems such as adverse drug reactions, complicated operations, and prolonged treatment periods (Urbanova et al., 2018).

Noninvasive brain stimulation (NIBS) therapy has become a hot topic of research for improving cognitive impairment after stroke (Li H. et al., 2021; Kim et al., 2022). NIBS mainly consists of transcranial electrical stimulation (TES) and transcranial magnetic stimulation (TMS). TES works by placing the positive and negative electrodes on the scalp surface and applying a current of 1–2 milliamps. This current alters the resting potential of the nerve cell membrane, lowers or raises the activation threshold of the neuron, and regulates the neuron's activity (Liu et al., 2018). TES primarily includes transcranial direct current stimulation (tDCS), transcranial alternating current stimulation (tACS) and transcranial random noise stimulation (tRNS). At present, there is more evidence of high-quality clinical studies on tDCS in rehabilitating cognitive impairment after stroke. However, the clinical application of tACS and tRNS is still in its infancy. The tDCS mode of action includes anodal tDCS stimulation alone, cathodal tDCS stimulation alone, and bilateral simultaneous anodal and cathodal tDCS stimulation (Solomons and Shanmugasundaram, 2019; Bhattacharya et al., 2022). TMS works by a coil placed on the scalp to transmit short pulses of current, creating a pulsed magnetic field (Hernandez-Pavon and Harvey, 2019). This magnetic field causes an induced current to form in the cerebral cortex at the site of stimulation, which alters the membrane potential of nerve cells and affects metabolism and associated electrophysiological activity in the brain (Klomjai et al., 2015). TMS stimulation modes primarily includes repetitive TMS (rTMS) and theta burst stimulation (TBS). According to different frequency parameters, rTMS can be divided into high-frequency rTMS (3–20 Hz) and low-frequency rTMS (≤1Hz); TBS can be divided into intermittent TBS (iTBS) and continuous TBS (cTBS) (Smith and Stinear, 2016).

Previous studies have shown that NIBS is important in rehabilitating post-stroke cognitive impairment. Kang et al. (2009) found that anodal tDCS stimulation significantly improved attentional function in patients with PSCI. Smirni et al. (2015) found that cathodal tDCS of the right dorsolateral prefrontal cortex (DLPFC) can improve recognition memory in healthy people. Shaker et al. (2018) treated PSCI patients with bilateral tDCS, and the patients showed significant improvements in attention and logical reasoning. Tsai et al. (2020) found significant improvements in attention and delayed memory after applying 5 Hz rTMS to patients with PSCI. Kim et al. (2018) found that 0.9 Hz rTMS improved cognitive function in stroke patients. However, the sample sizes of individual studies were minor, inclusion criteria and study methods varied, and there was no evidence of a difference in treatment effects between the two NIBS modalities.v This is highly detrimental to developing the clinical practice of NIBS for post-stroke cognitive impairment. Therefore, in this study, the efficacy of different NIBS stimulation techniques was evaluated and ranked according to the pathophysiological basis of PSCI using a network meta-analysis (NMA) to find the optimal neurostimulation protocol for patients with PSCI and to provide an evidence-based basis for clinical treatment decisions.

This systematic evaluation program has completed registration in the PROSPERO database (CRD42022372354).

## Materials and methods

### Search strategy

Computer searches of PubMed, Web of Science, Cochrane Library, Embase, China National Knowledge Infrastructure (CNKI), China Science and Technology Journal Database (VIP), Chinese Biomedical literature Service System (SinoMed), and Wanfang Database were conducted using a combination of free words and subject terms. The search was conducted from the database creation date to 27 November 2022. The search formula was (stroke OR cerebrovascular OR hemiplegia OR cerebral hemorrhage OR cerebral infarction OR cerebral stroke OR acute stroke) AND (noninvasive brain stimulation OR transcranial electrical stimulation OR transcranial direct current stimulation OR transcranial alternating current stimulation OR transcranial random noise stimulation OR transcranial magnetic stimulation OR repetitive transcranial magnetic stimulation OR theta burst stimulation OR intermittent theta burst stimulation OR continuous theta burst stimulation OR TES OR tDCS OR tACS OR tRNS OR TMS OR rTMS OR TBS OR iTBS OR cTBS) AND (cognitive dysfunction OR cognitive impairment OR cognition disorders) AND (randomized controlled trial OR random OR controlled trials OR RCT). After each of the two researchers (YW, RW) had completed the search independently, the results were cross-checked. In disagreement, the decision was discussed with a third researcher (NX).

### Inclusion criteria

1. Population: patients with a precise clinical diagnosis of hemorrhagic stroke or ischemic stroke, with no restrictions on nationality, gender, age, or educational background, had significant cognitive impairment.
2. Intervention: NIBS.
3. Comparison: sham-NIBS.
4. Outcome: montreal cognitive assessment (MoCA), mini-mental state examination (MMSE), modified barthel index (MBI), and functional independence measurement (FIM).
5. Study design: randomized controlled trial (RCT).

### Exclusion criteria

Animal experiments and repeat studies; interventions other than NIBS and conventional cognitive rehabilitation training were present in the experimental group; unavailability of full text; failure to extract outcome data; non-RCT studies such as self-control and case-control studies.

### Data extraction

Export the titles and abstracts of the retrieved documents and use Endnote 20 to eliminate duplicates. An initial screening of the literature was completed by browsing through the titles and abstracts. The literature was downloaded and read carefully to identify literature for inclusion based on inclusion and exclusion criteria. The above screening process was carried out independently by two researchers (RW, WZ), and the results were cross-checked. In disagreement, the decision was discussed with a third researcher (NX). Data were extracted from the literature, including first author, year, sample size, gender, age, course of disease, intervention, stimulation site, intervention length, evaluation time, and outcome indicators. Data were recorded using an Excel spreadsheet. Outcome data (mean ± standard deviation [SD]) for the final included literature were approximated according to the formulae in the Cochrane Handbook for Systematic Reviews of Interventions (Higgins et al., 2022) to eliminate potential differences in patients at baseline further. The value of the correlation coefficient (Corr) was 0.5.


$$\begin{array}{l} {\text{Mean}}_{\text{change}}{\text{ = Mean}}_{\text{final}}\text{ − }{\text{Mean}}_{\text{baseline}}\\ {\text{SD}}_{\text{change}}\\ =\sqrt{{\text{SD}}_{\text{baseline}}^{2}+{\text{SD}}_{\text{final}}^{\text{2}}\;-\;(2\times \text{Corr }\times {\text{SD}}_{\text{baseline }}\times \;{\text{SD}}_{\text{final}})} \end{array}$$

### Quality assessment

The risk of bias in the included literature was assessed using the Cochrane Risk Assessment Scale. The scale consists of seven components: random sequence generation, allocation concealment, blinding of investigators and subjects, blinded assessment of study results, completeness of outcome data, selective reporting of study results, and other biases. Risk levels were determined using “low risk of bias”, “high risk of bias”, and “uncertain risk of bias”. The quality of the included literature was assessed using the physiotherapy evidence database (PEDro) scale. The scale consists of 11 items: eligibility criteria were specified, random participant, allocation concealed, allocation groups similar at baseline, subject blinding, therapist blinding, assessor blinding, <15% dropout, intention to treat analysis, statistical comparisons between groups, point measures, and variability data. The first item was not scored, and the remaining ten were answered as yes (score = 1) or no (score = 0). A score out of 10 was assigned, with ≥7 being high quality, 5-6 being moderate quality, and ≤4 being low quality. The quality assessment was carried out independently by two researchers (YW, RW), and the results were cross-checked. In disagreement, the decision was discussed with a third researcher (NX).

### Statistical analysis

#### Meta-analysis

A standard meta-analysis of study data for each outcome indicator was performed using RevMan 5.4 software. *I*^2^ statistics and Cochrane's Q test were used to assess heterogeneity among included studies. If *I*^2^ ≤ 50% and *P* ≥ 0.1, there was considered no significant heterogeneity between the included studies, and the data were analyzed using a fixed effects model. If *I*^2^ > 50% and *P* < 0.1, significant heterogeneity was considered between the included studies. A random-effects model was used to analyze the data, and subgroup and sensitivity analyses were used to identify sources of heterogeneity. The outcome indicators in this study are continuous variables, and the assessment methods used may differ for each study. The effect sizes were expressed as standardized mean differences (SMD) with 95% confidence intervals (CI). If *P* ≤ 0.05, the combined statistic of multiple studies is significant. If *P* > 0.05, multiple studies' combined statistic is insignificant.

#### Network meta-analysis

NMA was performed using the network and mvmeta packages in State 14.0 according to the Bayesian framework. Evidence network plots were drawn presenting direct comparisons or indirect comparisons of relationships between different interventions. The dots in the graph represent interventions, with larger dots indicating more patients using the intervention. A straight line indicates that a direct comparison exists between two interventions. The thickness of the line segment represents the number of studies with direct comparisons. The surface under the cumulative ranking curve (SUCRA) is used to express the ranking probability (0% ≤ SUCRA ≤ 100%). A larger SUCRA for an intervention indicates that the intervention is more effective.

#### Additional analyses and small study effects

Subgroup analysis of different outcome indicators based on the timing of the intervention to determine the optimal intervention period. Comparison-adjusted funnel plots for NMA were drawn to determine the presence of small sample effects and publication bias based on symmetry.

## Results

### Study selection

**Figure 1:**
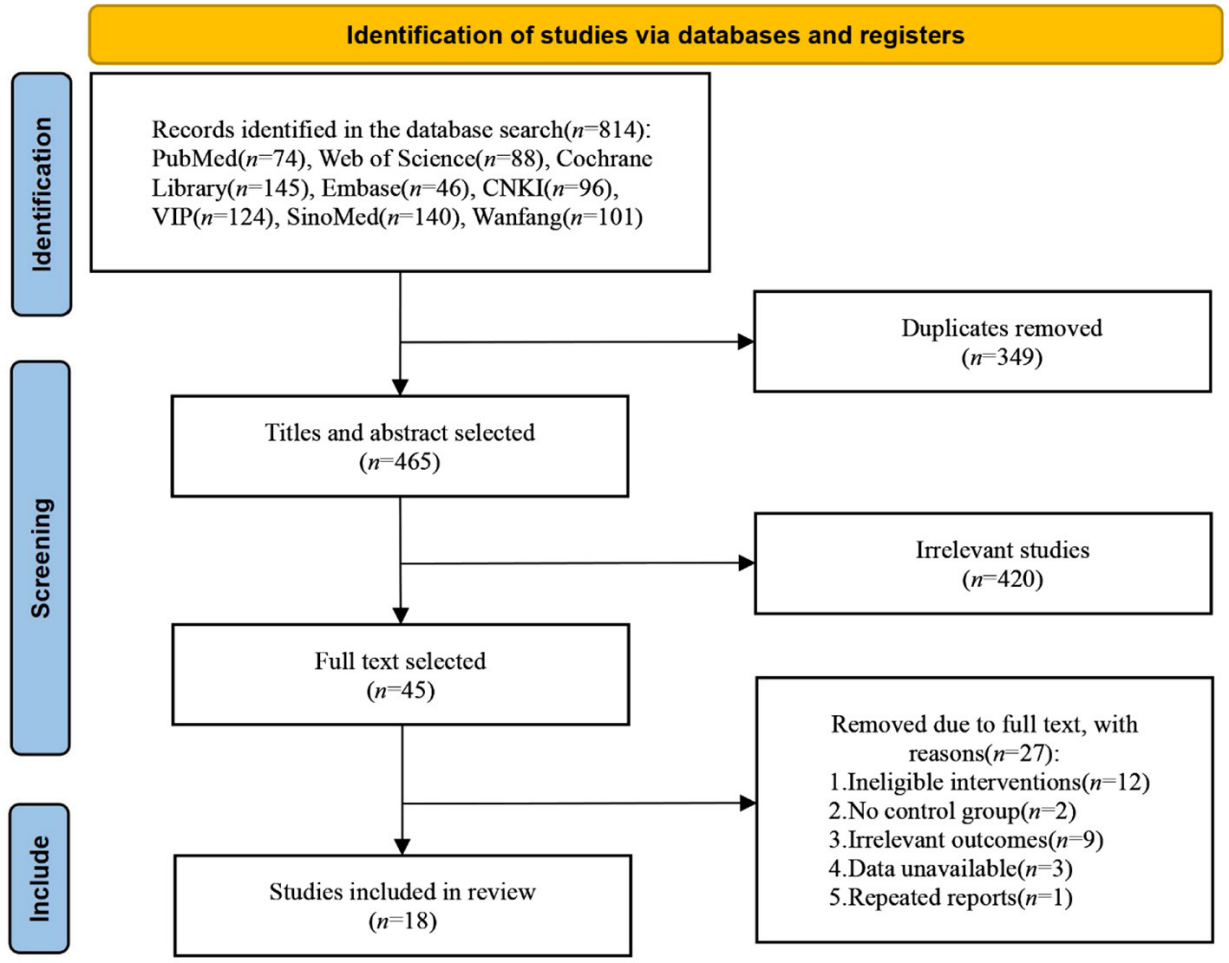
Screening process of literature selection.

A total of 814 studies were obtained, including 353 in English and 461 in Chinese. 18 studies (Kim et al., 2010; Lu et al., 2015; Yun et al., 2015; Shaker et al., 2018; Yin et al., 2018; Zeng et al., 2019; Zhang and Zou, 2019; Liu et al., 2020, 2021; Li Y. et al., 2020; Ma et al., 2020; Ai et al., 2021; Li H. et al., 2021; Zhang et al., 2021; Chen et al., 2022; Ko et al., 2022; Li W. et al., 2022; Yan et al., 2022) were finally included, including 9 in English (Kim et al., 2010; Lu et al., 2015; Yun et al., 2015; Shaker et al., 2018; Liu et al., 2020; Li Y. et al., 2020; Li H. et al., 2021; Ko et al., 2022; Li W. et al., 2022) and 9 in Chinese (Yin et al., 2018; Zeng et al., 2019; Zhang and Zou, 2019; Ma et al., 2020; Ai et al., 2021; Liu et al., 2021; Zhang et al., 2021; Chen et al., 2022; Yan et al., 2022). The literature selection process is shown in Figure 1. 809 patients were included in the 18 studies, including 418 in the NIBS group and 391 in the sham stimulation group. The essential characteristics of the included literature are shown in Table 1.

**Table 1:** Characteristics of included studies.

**Reference**	**Sample size (E/C)**	**Gender (male/** **female)**	**Age (E/C, year)**	**Course of disease (E/C, day/week/** **month)**	**Intervention**	**Stimulation**	**Intervention length**	**Evaluation**	**Outcome**
Yun et al. (2015)	15/15/15	6/9; 7/8; 7/8	(60.9 ± 12.9)/(58.9 ± 15.0)/(68.5 ± 14.6)	(42.2 ± 31.9)/(38.1 ± 27.0)/(39.5 ± 29.6) d	2.0 mA tDCS	Anode: left fronto-temporal area/anode: right fronto-temporal area	30 min/d, 5 d/wk, 3 wks	Before the intervention; after 3 wks	K-MMSE, K-MBI
Shaker et al. (2018)	20/20	Not described	(54.45 ± 4.68)/(53.05 ± 6.32)	(14.05 ± 1.53)/(16.55 ± 2.78) m	2.0mA tDCS	Anode: left or right DLPFC cathode: contralateral area	30 min/d, 3d/wk, 1 mo	Before the intervention; after 1 mo	FIM
Zeng et al. (2019)	15/15	9/6; 11/4	(56.21 ± 9.11)/(53.14 ± 7.12)	(41.29 ± 10.37)/(43.36 ± 12.17) d	2.0 mA tDCS	Left DLPFC	20 min/d, 5 d/wk, 4 wks	Before the intervention; after 4 wks	MoCA, MMSE
Ai et al. (2021)	14/13/14	11/3; 10/3; 10/4	(61.64 ± 10.33)/(61.36 ± 8.51)/(58.77 ± 9.61)	(7.75 ± 6.66)/(4.77 ± 2.19)/(6.77 ± 5.70) w	2.0 mA tDCS	Anode: left DLPFC cathode: right supraorbital area; tDCS treatment and conventional rehabilitation at the same time/separate tDCS treatment and conventional rehabilitation	30 min/d, 5d/wk, 2wks	Before the intervention; after 2 wks	MoCA, MBI
Liu et al. (2021)	20/20	12/8; 7/13	(63.72 ± 8.41)/(60.06 ± 8.26)	2.5(1, 3)/2.5(2, 4) m	2.0 mA tDCS	Anode: left DLPFC cathode: right DLPFC	20 min/d, 5 d/wk, 4 wks	Before the intervention; after 4 wks	MMSE
Chen et al. (2022)	36/36	18/18; 19/17	(64.01 ± 5.71)/ (63.58 ± 5.48)	(37.18 ± 10.52)/ (36.74 ± 10.23) d	1.5–2.0 mA tDCS	Anode: C3 or C4 of the primary motor cortex cathode: bilateral supraorbital area	20 min/d, 5 d/wk, 4 wks	Before the intervention; after 4 wks	MoCA, MMSE
Yan et al. (2022)	30/30	16/14; 17/13	(56.07 ± 8.52)/(57.40 ± 7.88)	(39.87 ± 12.67)/(38.90 ± 13.26) d	2.0 mA tDCS	Anode: the affected side of DLPEC	20 min/d, 5 d/wk, 4 wks	Before the intervention; after 4 wks	MoCA
Ko et al. (2022)	12/14	4/8; 8/6	(61.25 ± 12.85)/(57.86 ± 10.04)	Not described	2.0 mA RS-tDCS	Anode: left DLPFC cathode: right supraorbital area	30 min/d, 5 d/wk, 4 wks	Before the intervention; after 4 wks	K-MoCA
Liu et al. (2020)	29/29	10/19; 16/13	(58.55 ± 6.24)/(57.69 ± 7.25)	(8.79 ± 1.84)/(8.62 ± 1.84) m	10 Hz TMS, 90% RMT	Left DLPFC	5 d/wk, 4 wks	Before the intervention; after 4 wks	MMSE
Yin et al. (2018)	12/13	11/1; 12/1	(58.58 ± 11.98)/(60.15 ± 10.29)	(59.83 ± 30.59)/ (56.15 ± 23.74) d	10 Hz rTMS, 80% RMT	Left DLPFC	20 min/d, 5 d/wk, 4 wks	Before the intervention; after 2 wks; after 4 wks	MoCA, MBI
Ma et al. (2020)	30/30	18/12; 17/13	(58.53 ± 13.63)/(59.20 ± 13.06	(2.47 ± 0.88)/(2.38 ± 0.86) m	10 Hz rTMS, 80% RMT	Left DLPFC	20 min/d, 5 d/wk, 4 wks	Before the intervention; after 4 wks	MoCA
Kim et al. (2010)	6/6/6	4/2; 2/4; 4/2	(53.5 ± 16.9)/(68.3 ± 7.4)/(66.8 ± 17.2)	(241.2 ± 42.5)/(404.4 ± 71.7)/(69.7 ± 39.0) d	10 Hz rTMS, 80% RMT/1Hz rTMS, 80% RMT	Left DLPFC	5 d/wk, 2wks	Before the intervention; after 2 wks	MBI
Zhang and Zou (2019)	30/30	20/10; 18/12	(58.44 ± 16.60)/(55.11 ± 18.03)	(46.83 ± 28.13)/ (49.00 ± 37.01) d	5 Hz rTMS, 80% RMT	Left DLPFC	20 min/d, 5 d/wk, 4 wks	Before the intervention; after 4 wks	MoCA, MBI
Li Y. et al. (2020)	14/14	Not described	(65.47 ± 3.68)/(64.53 ± 4.72)	(22.73 ± 8.05)/(19.13 ± 7.95) d	5 Hz rTMS, 100% RMT	Left DLPFC	5 d/wk, 3 wks	Before the intervention; after 3 wks	MoCA, MMSE
Lu et al. (2015)	19/21	12/7; 13/8	(42.5 ± 12.3)/(47.3 ± 11.8)	67 (30, 365)/56 (30, 296) d	1 Hz rTMS, 100% RMT	Right DLPEC	5 d/wk, 4 wks	Before the intervention; after 4 wks	MoCA
Li H. et al. (2021)	33/32	21/12; 19/13	(61.79 ± 5.51)/(59.47 ± 6.7)	(28.64 ± 12.60)/(27.78 ± 11.01) d	1 Hz rTMS, 90% RMT	Contralateral DLPEC	20 min/d, 5 d/wk, 4 wks	Before the intervention; after 4 wks	MoCA, MBI
Zhang et al. (2021)	21/22	15/6; 14/8	(60.67 ± 9.53)/(58.95 ± 7.88)	(51.90 ± 21.90)/49.50 ± 29.39) d	1 Hz rTMS, 90% RMT	Contralateral DLPEC	20 min/d, 5 d/wk, 4 wks	Before the intervention; after 4 wks	MoCA, MMSE
Li W. et al. (2022)	28/30	16/12; 18/12	69.5 (60.0,78.0)/66.0 (53.0,75.0)	25(17,30)/ 25(18,30) d	iTBS, three continuous pulses at 50 Hz repeated at 5 Hz (2s on, 8s off) for a total of 192 s and 600 pulses	Left DLPFC	5 d/wk, 2wks	Before the intervention; after 2 wks	MMSE

Data presented as mean ± SD or median (interquartile range, IQR); E, experiment group; C, control group; RMT, resting motor threshold.

### Quality assessment

**Figure 2:**
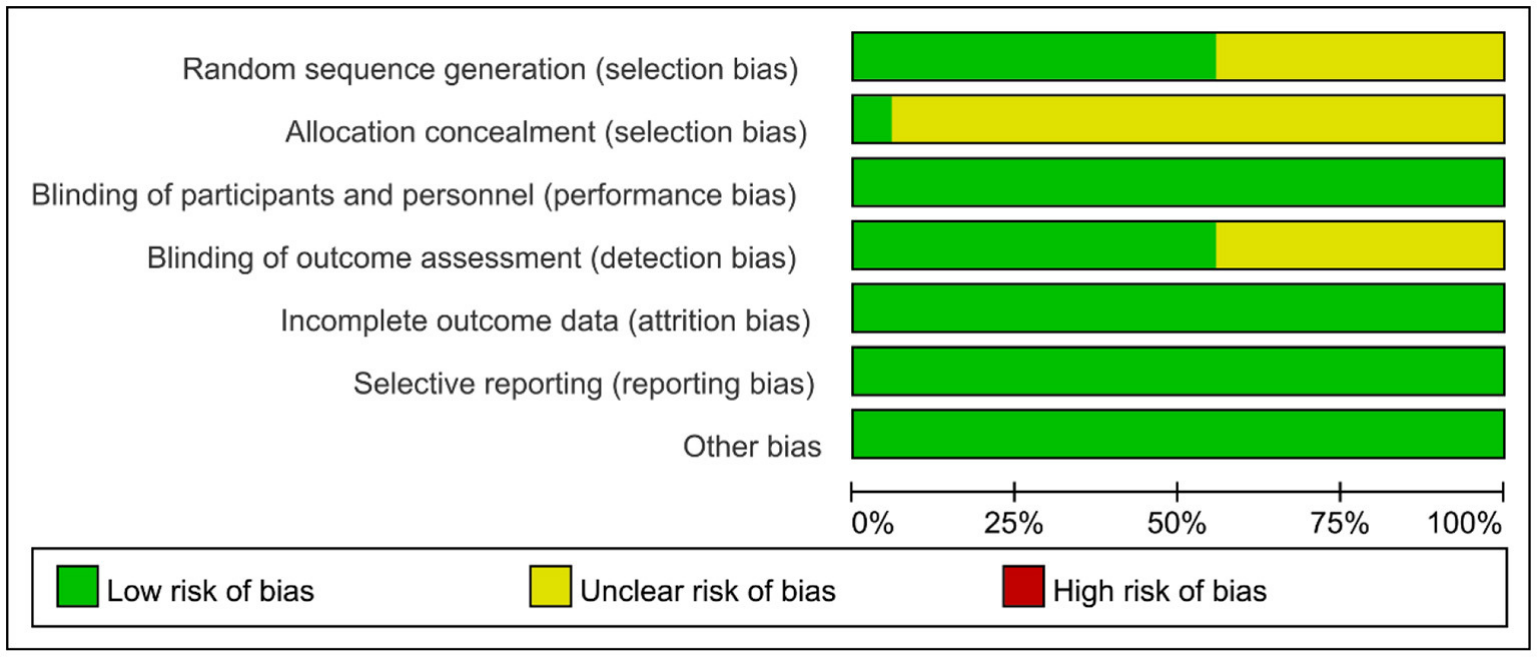
Risk assessment of bias.

Of the 18 studies, eight (Lu et al., 2015; Yin et al., 2018; Zeng et al., 2019; Zhang and Zou, 2019; Ai et al., 2021; Li H. et al., 2021; Liu et al., 2021; Zhang et al., 2021) used the number table method to generate random sequences, two (Liu et al., 2020; Ko et al., 2022) used a computer for randomized assignment. The remaining eight mentioned “random grouping” but did not specify the randomization method. Only one study used sealed opaque envelopes for allocation concealment (Liu et al., 2020). All studies were blinded to treatment participants. Ten studies (Kim et al., 2010; Yun et al., 2015; Yin et al., 2018; Zhang and Zou, 2019; Liu et al., 2020, 2021; Li Y. et al., 2020; Ai et al., 2021; Ko et al., 2022; Li W. et al., 2022) were blinded to assessors. The results of all studies were complete and not selectively reported. The distribution of the risk of bias across studies is shown in Figure 2. The 18 studies were all high quality, with a mean score of 8 (Table 2).

**Table 2:** Physiotherapy evidence database scores of the included studies.

**Study**	**1**	**2**	**3**	**4**	**5**	**6**	**7**	**8**	**9**	**10**	**11**	**Total**	**Quality level**
Yun et al. (2015)	Yes	1	0	1	1	1	1	1	1	1	1	9/10	High
Shaker et al. (2018)	Yes	1	0	1	1	0	0	1	1	1	1	7/10	High
Zeng et al. (2019)	Yes	1	0	1	1	0	0	1	1	1	1	7/10	High
Ai et al. (2021)	Yes	1	0	1	1	0	1	1	1	1	1	8/10	High
Liu et al. (2021)	Yes	1	0	1	1	0	1	1	1	1	1	8/10	High
Chen et al. (2022)	Yes	1	0	1	1	0	0	1	1	1	1	7/10	High
Yan et al. (2022)	Yes	1	0	1	1	0	0	1	1	1	1	7/10	High
Ko et al. (2022)	Yes	1	0	1	1	1	1	1	1	1	1	9/10	High
Liu et al. (2020)	Yes	1	1	1	1	1	1	1	1	1	1	10/10	High
Yin et al. (2018)	Yes	1	0	1	1	0	1	1	1	1	1	8/10	High
Ma et al. (2020)	Yes	1	0	1	1	0	0	1	1	1	1	7/10	High
Kim et al. (2010)	Yes	1	0	1	1	1	1	1	1	1	1	9/10	High
Zhang and Zou (2019)	Yes	1	0	1	1	0	1	1	1	1	1	8/10	High
Li Y. et al. (2020)	Yes	1	0	1	1	1	1	1	1	1	1	9/10	High
Lu et al. (2015)	Yes	1	0	1	1	1	0	1	1	1	1	8/10	High
Li H. et al. (2021)	Yes	1	0	1	1	0	0	1	1	1	1	7/10	High
Zhang et al. (2021)	Yes	1	0	1	1	0	0	1	1	1	1	7/10	High
Li W. et al. (2022)	Yes	1	0	1	1	1	1	1	1	1	1	9/10	High

### Meta-analysis

#### Cognitive functions

##### MoCA

**Figure 3:**
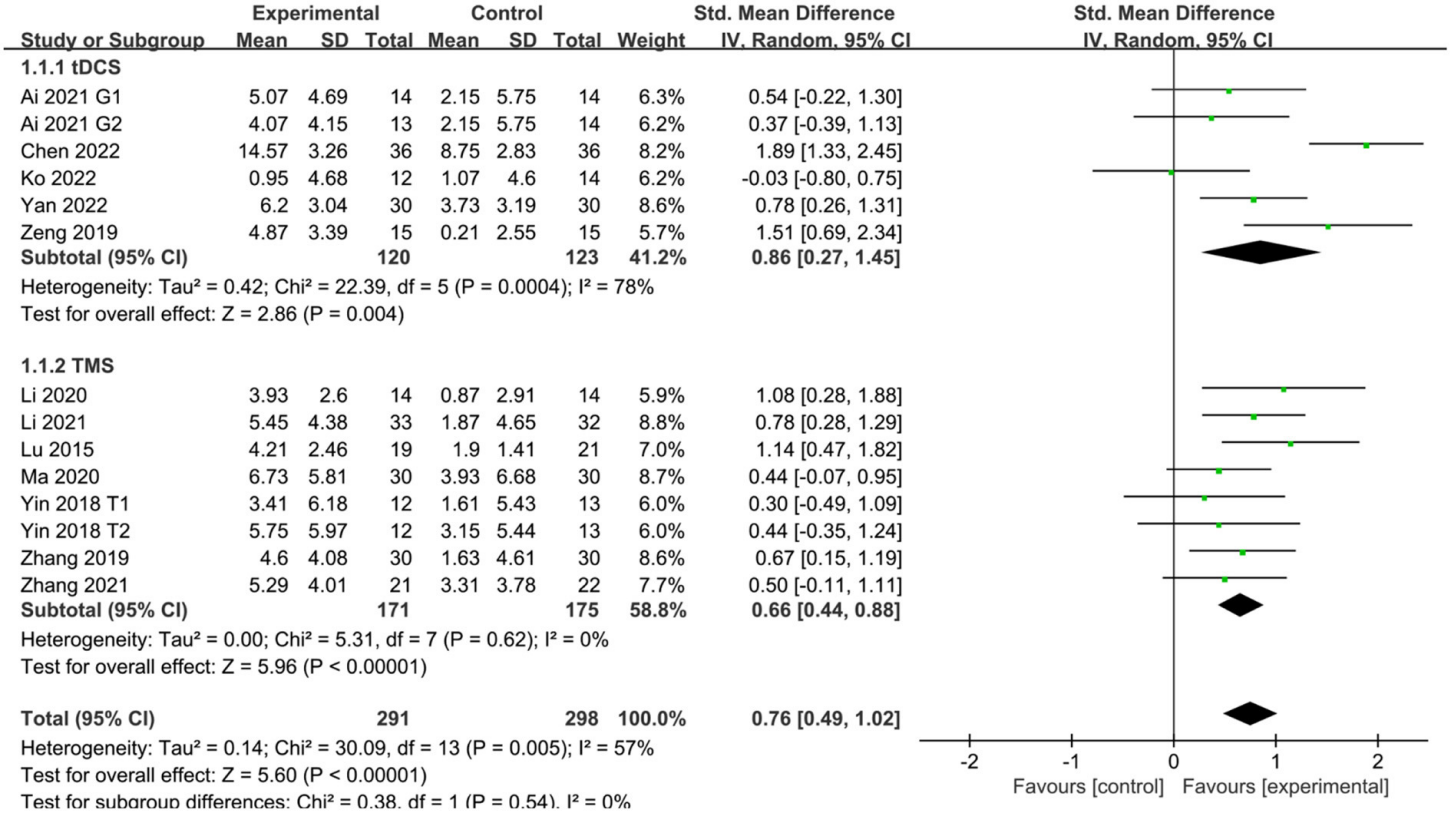
Effect of NIBS on MoCA score in PSCI patients.

Twelve studies (Lu et al., 2015; Yin et al., 2018; Zeng et al., 2019; Zhang and Zou, 2019; Li Y. et al., 2020; Ma et al., 2020; Ai et al., 2021; Li H. et al., 2021; Zhang et al., 2021; Chen et al., 2022; Ko et al., 2022; Yan et al., 2022) reported MoCA scores in patients with PSCI after treatment. The results showed that MoCA scores were better in the NIBS group than in the sham stimulation group, with a statistically significant difference (SMD = 0.76, 95% CI 0.49–1.02, *P* < 0.05). Subgroup analysis showed that both the tDCS and TMS groups were more effective than the sham stimulation group, with a statistically significant difference (SMD = 0.86, 95% CI 0.27–1.45, *P* < 0.05 and SMD = 0.66, 95% CI 0.44–0.88, *P* < 0.05) (Figure 3).

##### MMSE

**Figure 4:**
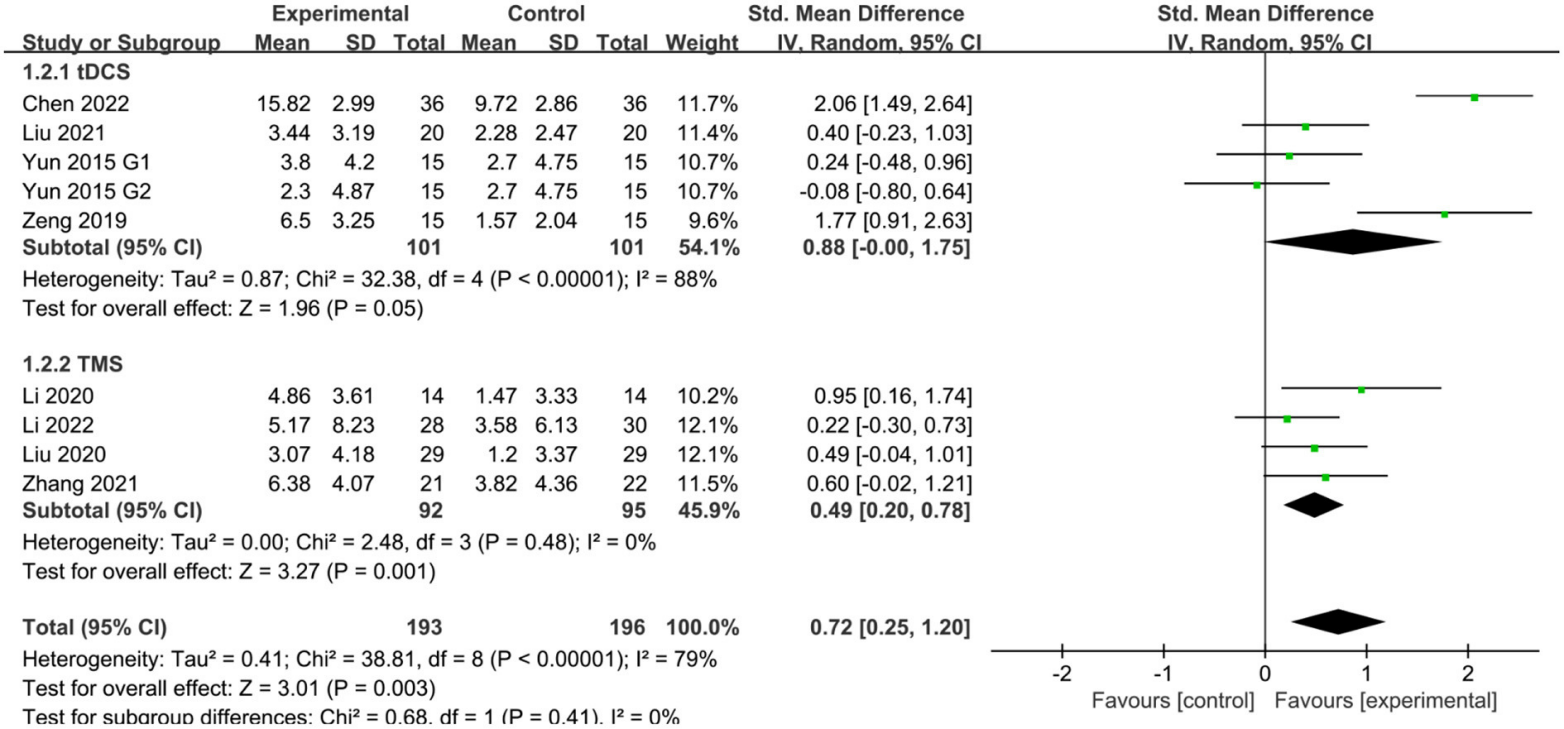
Effect of NIBS on MMSE score in PSCI patients.

Eight studies (Yun et al., 2015; Zeng et al., 2019; Liu et al., 2020, 2021; Li Y. et al., 2020; Zhang et al., 2021; Chen et al., 2022; Li W. et al., 2022) reported MMSE scores in patients with PSCI after treatment. The results showed that MMSE scores were better in the NIBS group than in the sham stimulation group, with a statistically significant difference (SMD = 0.72, 95% CI 0.25–1.20, *P* < 0.05). Subgroup analysis showed that both the tDCS and TMS groups were more effective than the sham stimulation group, with a statistically significant difference (SMD = 0.88, 95% CI −0.00–1.75, *P* = 0.05 and SMD = 0.49, 95% CI 0.20–0.78, *P* < 0.05) (Figure 4).

### Activities of daily living

**Figure 5:**
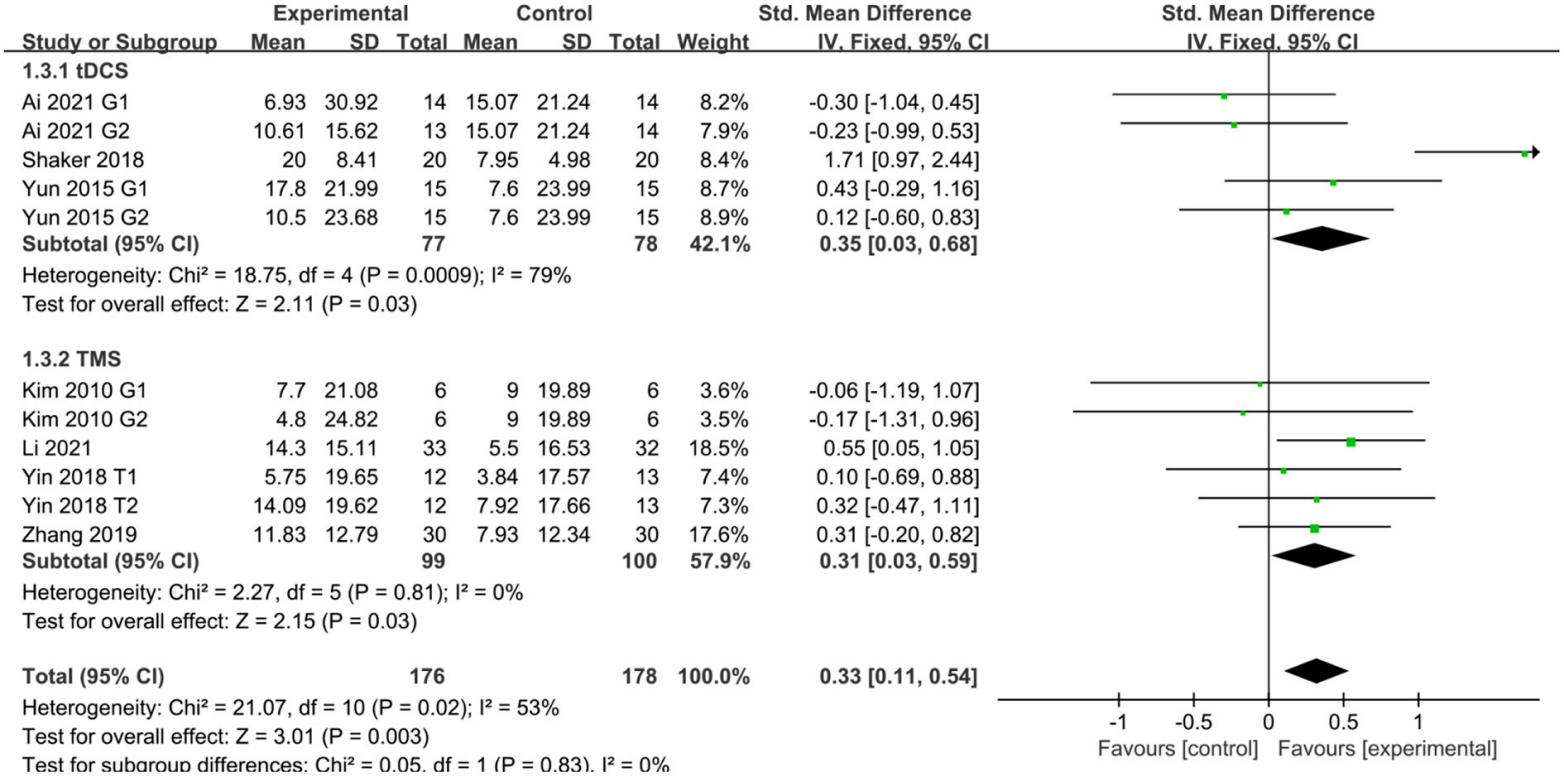
Effect of NIBS on MBI and FIM score in PSCI patients.

Seven studies (Kim et al., 2010; Yun et al., 2015; Shaker et al., 2018; Yin et al., 2018; Zhang and Zou, 2019; Ai et al., 2021; Li H. et al., 2021) reported activities of daily living in patients with PSCI after treatment. Six studies (Kim et al., 2010; Yun et al., 2015; Yin et al., 2018; Zhang and Zou, 2019; Ai et al., 2021; Li H. et al., 2021) used MBI to assess patients and one study (Shaker et al., 2018) used FIM to assess patients. The results showed that activities of daily living were better in the NIBS group than in the sham stimulation group, with a statistically significant difference (SMD = 0.33, 95% CI 0.11–0.54, *P* < 0.05). Subgroup analysis showed that both the tDCS and TMS groups were more effective than the sham stimulation group, with a statistically significant difference (SMD = 0.35, 95% CI 0.03–0.68, *P* < 0.05 and SMD = 0.31, 95% CI 0.03–0.59, *P* < 0.05) (Figure 5).

### Network meta-analysis

#### Cognitive functions

##### MoCA

**Figure 6:**
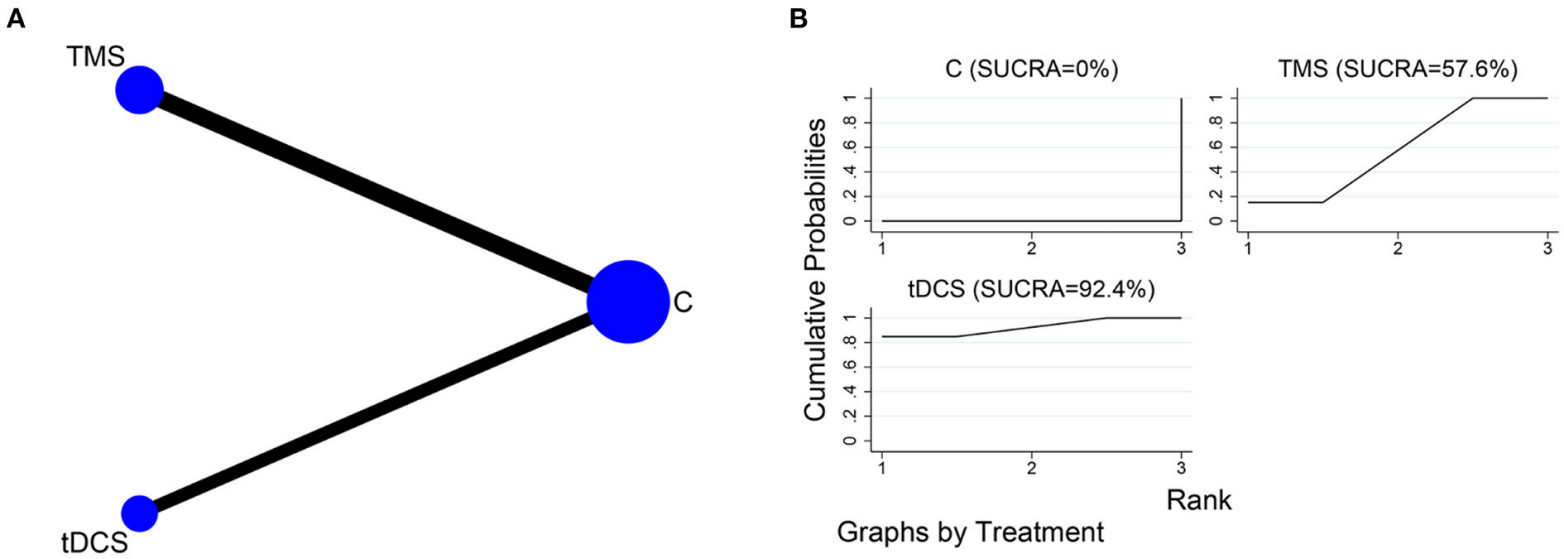
NMA of MoCA scores at different NIBS. **(A)** Network plot; **(B)** SUCRA plot.

The network relationships for the different NIBS, using MoCA as the outcome indicator, are shown in Figure 6A. Five of the included studies (Zeng et al., 2019; Ai et al., 2021; Chen et al., 2022; Ko et al., 2022; Yan et al., 2022) had tDCS as the intervention, and seven (Lu et al., 2015; Yin et al., 2018; Zhang and Zou, 2019; Li Y. et al., 2020; Ma et al., 2020; Li H. et al., 2021; Zhang et al., 2021) had TMS as the intervention. The SUCRA of different NIBS in improving MoCA scores were in the order of tDCS (SUCRA = 92.4%) and TMS (SUCRA = 57.6%) (Figure 6B).

##### MMSE

**Figure 7:**
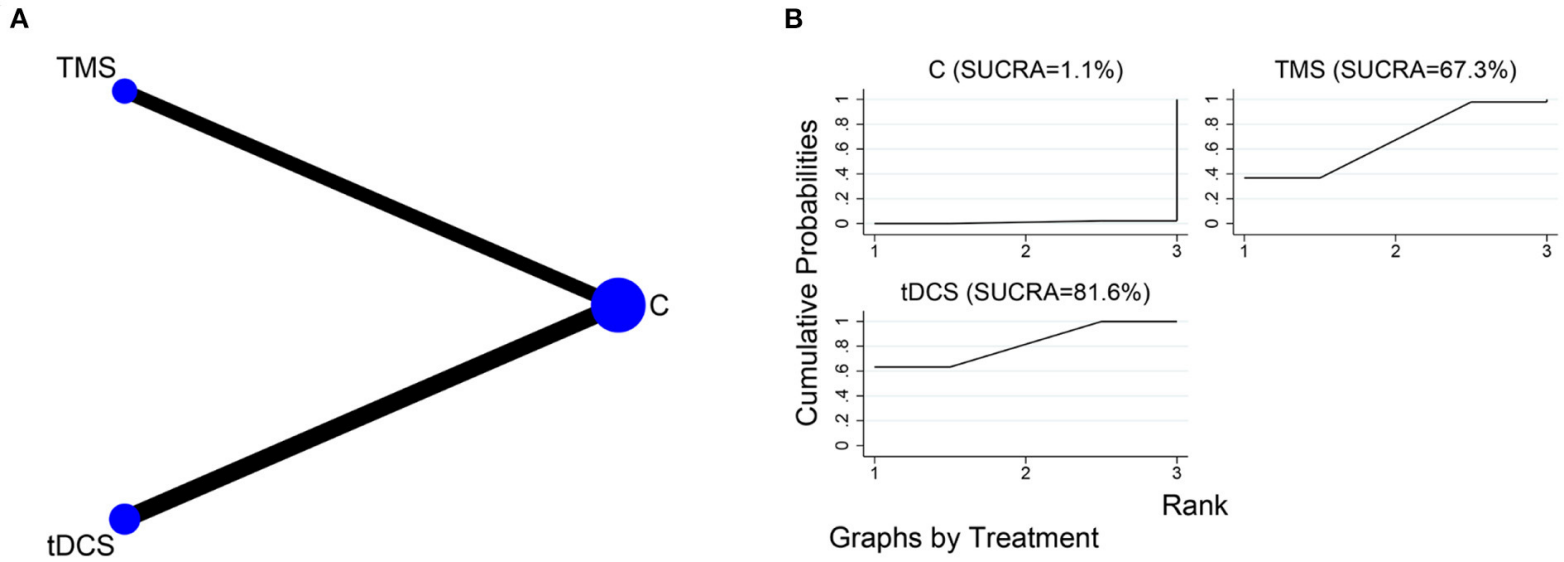
NMA of MMSE scores at different NIBS. **(A)** Network plot; **(B)** SUCRA plot.

The network relationships for the different NIBS, using MMSE as the outcome indicator, are shown in Figure 7A. Four of the included studies (Yun et al., 2015; Zeng et al., 2019; Liu et al., 2021; Chen et al., 2022) used tDCS as the intervention, and four (Liu et al., 2020; Li Y. et al., 2020; Zhang et al., 2021; Li W. et al., 2022) used TMS as the intervention. The SUCRA of different NIBS in improving MMSE scores were in the order of tDCS (SUCRA = 81.6%) and TMS (SUCRA = 67.3%) (Figure 7B).

### Activities of daily living

**Figure 8:**
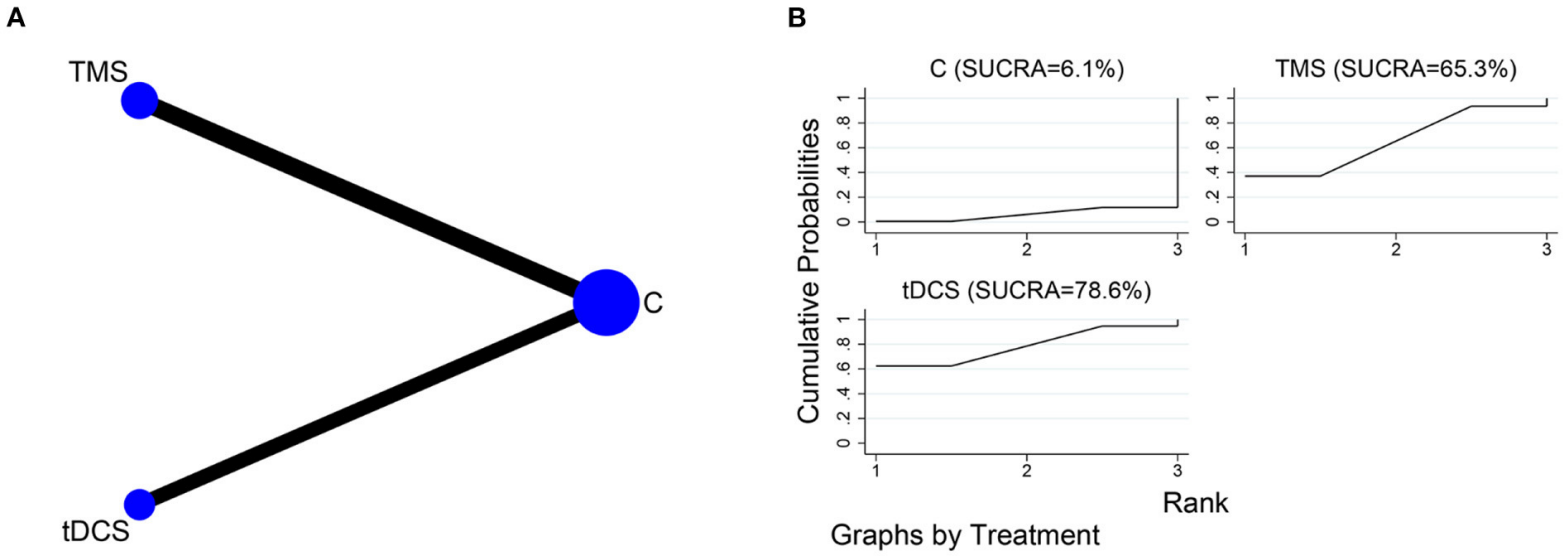
NMA of MBI and FIM scores at different NIBS. **(A)** Network plot; **(B)** SUCRA plot.

The network relationships for the different NIBS, using MBI and FIM as the outcome indicator, are shown in Figure 8A. Three of the included studies (Yun et al., 2015; Shaker et al., 2018; Ai et al., 2021) used tDCS as the intervention, and four (Kim et al., 2010; Yin et al., 2018; Zhang and Zou, 2019; Li H. et al., 2021) used TMS as the intervention. The SUCRA of different NIBS in improving activities of daily living were in the order of tDCS (SUCRA = 78.6%) and TMS (SUCRA = 65.3%) (Figure 8B).

### Adverse reaction

Six studies (Lu et al., 2015; Yin et al., 2018; Li Y. et al., 2020; Ai et al., 2021; Liu et al., 2021; Li W. et al., 2022) reported that a few patients experienced transient dizziness, pain, and pins and needles, and sneezing during treatment. The patients could be relieved after rest and did not affect the treatment. No adverse effects were reported in other studies.

### Subgroup analysis of outcomes

Subgroup analyses were conducted on the MoCA, MMSE, MBI, and FIM scores according to the length of the intervention. The results are shown in Table 3.

**Table 3:** Subgroup analysis of NIBS on PSCI patients.

**Subgroup analysis**		**Studies**	**SMD (95% CI)**	***P***	***X* ^2^**	***I*^2^ (%)**	**Tau^2^**
**MoCA**							
Intervention length	< 4w	4	0.44 [0.10, 0.79]	0.01	4.05	1%	0.00
	≥4w	9	0.89 [0.57, 1.22]	< 0.0001	21.59	63%	0.15
**MMSE**							
Intervention length	< 4w	3	0.29 [−0.08, 0.67]	0.28	3.80	21%	0.03
	≥4w	5	1.04 [0.34, 1.74]	0.003	24.97	84%	0.53
**MBI, FIM**							
Intervention length	< 4w	4	0.01 [−0.30, 0.32]	0.95	2.59	0%	0.00
	≥4w	4	0.69 [0.12, 1.27]	0.02	0.51	72%	0.24

### Sensitivity analysis

The meta-analysis results were analyzed for sensitivity using a one-by-one exclusion method, removing one study at a time. The results showed no significant change from the above results, indicating that the meta-analysis results were relatively stable.

### Publication bias

**Figure 9:**
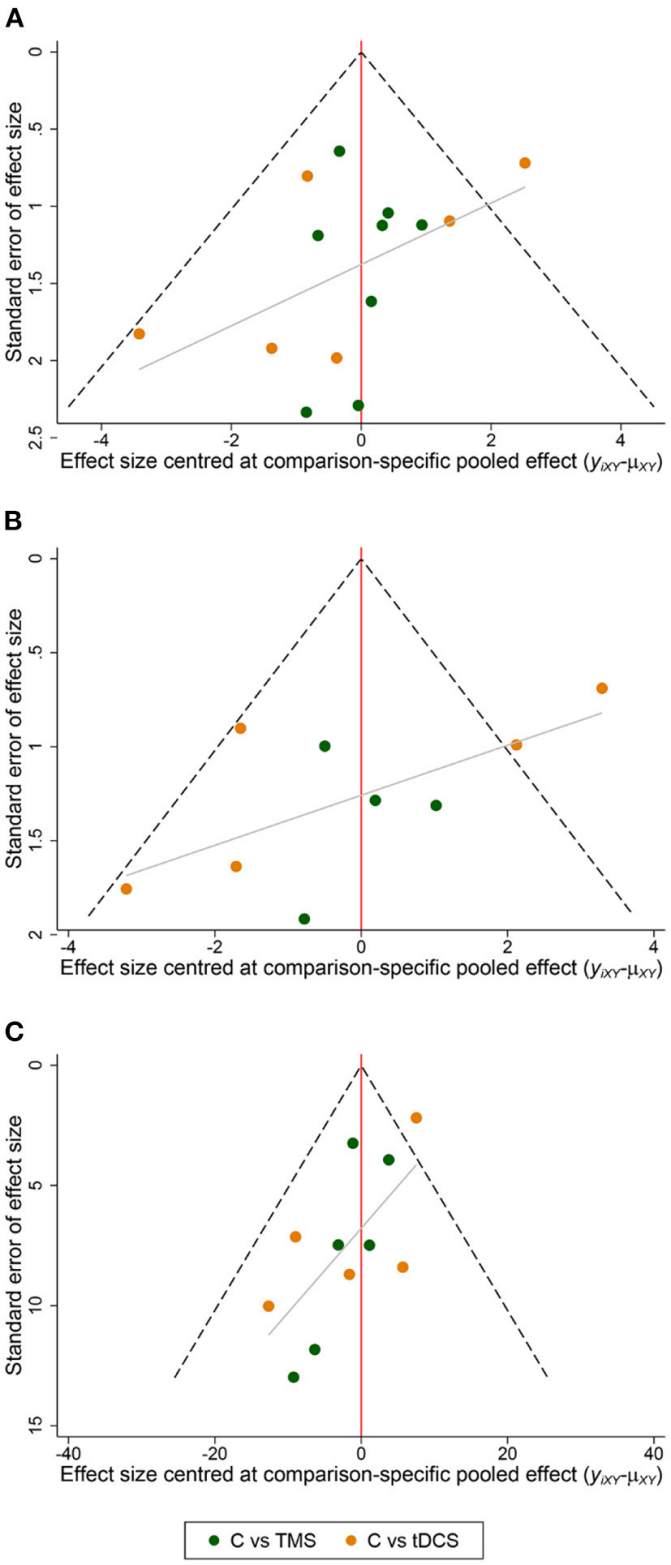
Funnel plot of the included studies. **(A)** MoCA; **(B)** MMSE; **(C)** MBI, FIM.

A funnel plot analysis of the included literature with MoCA, MMSE, MBI, and FIM as outcome indicators showed that the scatter was generally symmetrical, and the Meta-analysis results were reliable (Figure 9).

## Discussion

In the included studies, patients' cognitive function was assessed using MoCA and MMSE, and patient's ability to perform activities of daily living was assessed using MBI and FIM. The meta-analysis showed that both tDCS and TMS significantly improved the cognitive function and activities of daily living of PSCI patients compared to the control group. Network meta-analysis showed that tDCS appeared more effective than TMS for cognitive function and activities of daily living in PSCI patients. NIBS stimulation parameters and treatment duration are important factors that also influence efficacy. In the included literature, the parameters of tDCS were mainly 2.0 mA for 20–30 min; the TMS stimulation modality commonly used was rTMS, with low-frequency rTMS mainly at 1 Hz and high-frequency rTMS at 5 and 10 Hz for about 20 min. Better cognitive rehabilitation results were achieved with a total intervention time of NIBS above 4 weeks.

NIBS' current mechanism of action on improving cognitive function in patients with PSCI consists of three main aspects: first, by affecting cortical excitability; second, by improving neuroplasticity; third, by regulating cerebral blood flow. The theory of interhemispheric competition suggests that the mechanism of PSCI is the inability of the affected cerebral hemisphere to form a normal inhibitory effect on the healthy hemisphere, resulting in pathological excitation in the healthy hemisphere (Di Pino et al., 2014). NIBS primarily uses two treatment modalities, excitation of the affected hemisphere and inhibition of the healthy hemisphere (Li L. et al., 2021), thereby facilitating the recovery of cognitive function in patients with PSCI. Kenney-Jung et al. (2019) found that anodal tDCS stimulation increased the frequency of spontaneous firing in neuronal cells and increased cortical excitability; cathodal tDCS stimulation caused hyperpolarization of neuronal cell membranes and decreased cortical excitability. It was shown that high-frequency rTMS stimulation activates many voltage-gated channels, producing a depolarizing effect and increasing cortical excitability; low-frequency rTMS inhibits neuronal activity and reduces cortical excitability (Klomjai et al., 2015; Mikellides et al., 2021).

Stroke causes damage to synaptic signal transmission and synaptic structures. Studies have shown that synaptic damage in the hippocampus is associated with decreased spatial learning and memory function (Xu et al., 2018). Therefore, improving synaptic plasticity is also a meaningful way to treat cognitive impairment (Rolland et al., 2013). NIBS has been shown to improve synaptic plasticity, and this change may be related to both long-term potentiation and long-term depression (Huang et al., 2017; Jones, 2017; Cavaleiro et al., 2020). In addition, Monai et al. (2016) showed that tDCS can modulate synaptic plasticity by altering the concentration of calcium ions in astrocytes. Further studies have shown that tDCS stimulation can affect synaptic plasticity by altering the concentration of γ-aminobutyric acid secreted by astrocytes (Antonenko et al., 2017). Lenz et al. (2016) found that 10 Hz rTMS can affect synaptic excitability in the proximal dendrites of hippocampal CA1 pyramidal neurons. Li et al. (2019) found that 0.5 Hz rTMS increased the density of synaptic ultrastructure in the hippocampal CA1 region.

Cerebral vascular occlusion after stroke leads to tissue infarction and propagation of damage to adjacent cells, creating an ischemic semidark zone between the ischemic site and normal tissue. Reduced local blood flow to brain tissue in the focal and semidark areas leads to ischemic white matter lesions and cognitive impairment (Inaba et al., 2019). It has been demonstrated that NIBS has the effect of modulating cerebral blood flow, which improves cognitive function. Bragina et al. (2018) found that anodal tDCS induces the dilation of small arteries and modulates capillary blood flow velocity, leading to increased cerebral blood flow. Hara et al. (2017) identified that the degree of decreased cerebral perfusion on the affected side was reduced after patients received high-frequency rTMS; patients receiving low-frequency rTMS had reduced perfusion in the healthy hemisphere, reduced inhibition on the affected side, and increased cerebral blood flow on the affected side.

## Limitations

There are some limitations to this study. First, the total number of subjects included in the literature was small. Second, some literature does not hide the order of assignment and assessor blinding, which may lead to a potential risk of bias. Third, age differences in the study population and varying severity of illness may have impacted the rehabilitation outcomes. Fourth, the frequency and periodicity of interventions in the literature varied, which may have biased the study results. Fifthly, some literature had short treatment cycles, and most studies did not have a long-term follow-up after treatment.

## Conclusion

In summary, NIBS has shown promising results in improving patients' cognitive function and activities of daily living with PSCI. In the future, more extensive and rigorous double-blind randomized controlled trials are needed to explore the optimal stimulation parameters and intervention cycles for NIBS. The combination of NIBS and brain imaging technology should be enhanced, and in-depth mechanistic studies should be conducted to provide more reliable evidence-based medical evidence for clinical rehabilitation.

## Data availability statement

The original contributions presented in the study are included in the article/supplementary material, further inquiries can be directed to the corresponding author.

## Author contributions

YW designed and wrote this study. NX guided the methodology. RW reviewed the entire manuscript. YW and RW took part in the data selection and extraction. RW and WZ performed the statistical analysis and analyzed the data. All authors contributed to the article and approved the submitted version.
